# Two cases of a rare association: double aortic arch with tetralogy of Fallot

**DOI:** 10.1186/1749-8090-8-39

**Published:** 2013-03-07

**Authors:** Zhi-qiang Li, Ai-jun Liu, Gang Li, Yang Liu, Yao-bin Zhu, Ying-long Liu

**Affiliations:** 1 Pediatric Heart Center, Beijing Anzhen Hospital, Capital Medical University, Beijing, China; 2 Pediatric Heart Center, Beijing Anzhen Hospital, Capital Medical University, 2 Anzhen Rd, Beijing, 100029, China

**Keywords:** Double aortic arch, Tetralogy of Fallot, Congenital heart disease

## Abstract

Two cases of asymptomatic double aortic arch with tetralogy of Fallot are reported. One presented with a non-dominant left arch and left-sided descending thoracic aorta and the other with a non-dominant left arch, a right-sided descending thoracic aorta and a patent ductus arteriosus. One-stage operation was performed and both patients were discharged free of symptoms and signs related to the double aortic arch and tetralogy of Fallot after the operation. A preoperative recognition of DAA is important, especially in echocardiographic investigation.

## Background

Double aortic arch (DAA) associated with tetralogy of Fallot (TOF) is a rare congenital disorder. We reported two cases of asymptomatic double aortic arch associated with TOF. One-stage operation was performed successfully in both patients, and they were discharged free of symptoms and signs related to the DAA and TOF after the operation.

## Case presentation

### Patient 1

**Figure 1:**
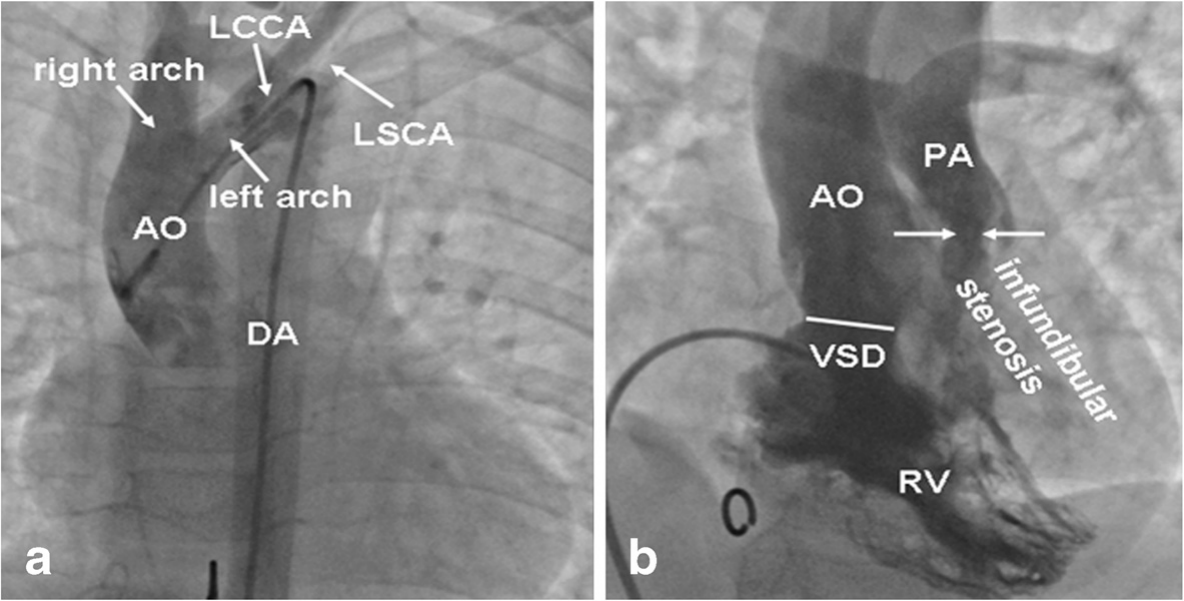
**Aortic root angiograms (a) and RV angiography (b) in anteroposterior.** AO = aorta, PA = pulmonary artery, DA = descending aorta, LCCA = left common carotid artery, LSCA = left subclavian artery, RV = right ventricle, VSD = ventricular septal defect.

An 8-year-old boy presented with exertional dyspnoea and mild cyanosis, but no history of stridor or dysphagia, was referred to us. Clinical features were as followings: 21 kilograms, 95 beats/min, and pulse oximeter oxygen saturation 87% on air. A harsh 3/6 ejection systolic murmur was heard in the third and fourth left intercostal space. The electrocardiogram revealed sinus rhythm with right-axis deviation and right ventricular hypertrophy. The X-ray displayed a typical coeur en sabot heart and normal lung fields. The echocardiography confirmed the diagnosis of TOF consisting of a 19 mm bidirectional subcrista ventricular septal defect (VSD), infundibular pulmonary artery stenosis with a gradient of 82 mmHg. Besides confirming TOF, a suspected DAA was diagnosed and the cardiac catheterization was requested for further analysis. After confirming TOF with mildly hypoplastic pulmonary annulus and pulmonary artery branches (McGoon ratio 1.7), a DAA with a non-dominant left arch and left-sided descending thoracic aorta was also detected: the left common carotid artery and the left subclavian artery arose individually from the smaller left component of the aortic arch, whereas the right common carotid artery and subclavian artery arose individually from the larger right aortic arch (Figure 1).

Surgical treatment, consisting of VSD closure, right ventricular out flow tract enlargement and resection of the distal end of the left aortic arch beyond left subclavian artery, was performed successfully. Postoperatively, the child was haemodynamically stable, and recovery was unremarkable. After a 46 months follow-up, the child was in New York Heart Association Class I and free of symptoms and signs related to the DAA and TOF. The pulse oximeter oxygen saturation on air was 96%. Follow-up cardiac catheterization or CT has not been performed so far to study the balance of circulation to either lung. However, follow-up echocardiograms have revealed satisfactory flow into the reconstructed pulmonary artery and without any gradients to descending aorta. The pressure gradient across pulmonary artery decreased to 20 mmHg after operation.

### Patient 2

**Figure 2:**
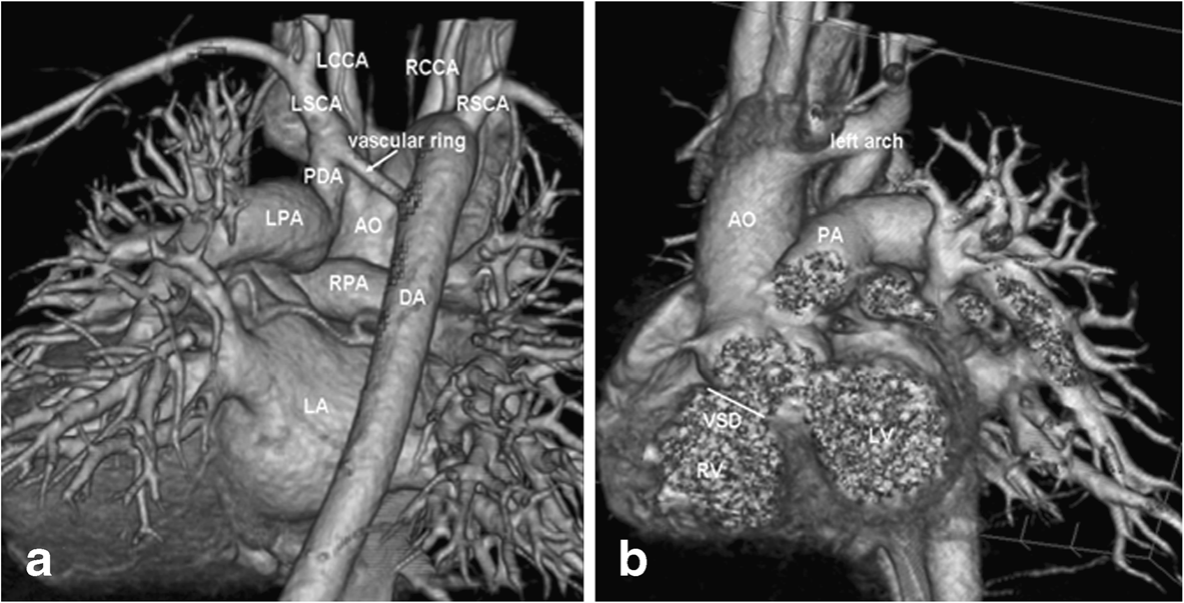
**3D-volume rendered image of the contrast enhanced multi-slice CT (a) in posteroanterior and (b) in anteroposterior.** AO = aorta, DA = descending aorta, PDA = patent ductus arteriosus, LPA = left pulmonary artery, RPA = right pulmonary artery, LCCA = left common carotid artery, LSCA = left subclavian artery, RCCA = right common carotid artery, RSCA = right subclavian artery, RV = right ventricle, LV = left ventricle, VSD = ventricular septal defect, LA = left atrium.

A 16-month-old girl presented with exertional dyspnoea and mild cyanosis, but no history of stridor or dysphagia, was referred to us. Clinical features were as followings: 10.5 kilograms, 108 beats/min, and pulse oximeter oxygen saturation 83% on air. A harsh 3/6 ejection systolic murmur was heard in the third and fourth left intercostal space, and a grade 3/6 systolic and diastolic murmurs was heard in the second left intercostal space. The electrocardiogram and X-ray display was same as patient 1. The echocardiography confirmed the diagnosis of TOF consisting of a 15 mm bidirectional subcrista VSD, infundibular pulmonary artery stenosis with a gradient of 103 mmHg and a suspected DAA. The computed tomography (CT) was requested further analysis. Besides confirming TOF with mildly hypoplastic pulmonary annulus and pulmonary artery branches (McGoon ratio 1.8), a DAA with a non-dominant left arch and right-sided descending thoracic aorta was also detected (Figure 2): the left common carotid artery and the left subclavian artery arose individually from the smaller left component of the aortic arch, whereas the right common carotid artery and subclavian artery arose individually from the larger right aortic arch. A patent ductus arteriosus connected left aortic arch with left pulmonary artery were also detected. The vascular ring surrounded the trachea and oesophagus, and the trachea was slightly compressed.

**Figure 3:**
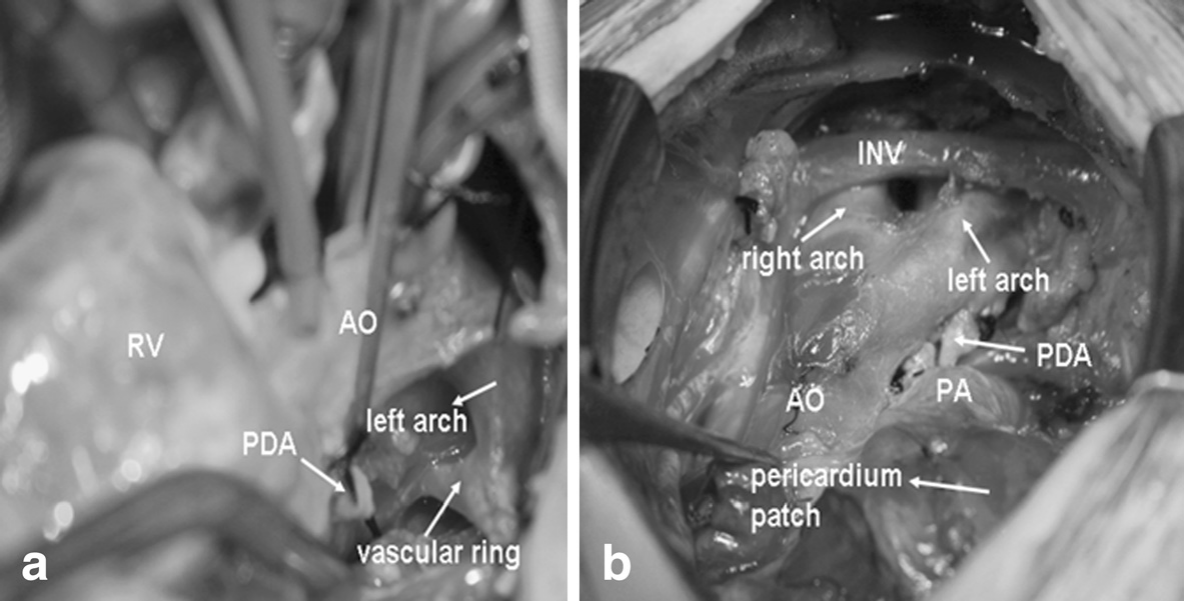
**Intraoperative (a) and postoperative (b) picture.** AO = aorta, PDA = patent ductus arteriosus, PA = pulmonary artery, RV = right ventricle, INV = innominate vein.

Surgical treatment, consisting of VSD closure, right ventricular out flow tract enlargement, resection of patent ductus arteriosus and resection of the distal end of the left aortic arch beyond left subclavian artery, was performed and the mild upper-airway obstruction was successfully relieved (Figure 3). Postoperatively, the child was haemodynamically stable, and recovery was unremarkable. After a 14 months follow-up, the child was in New York Heart Association Class I and free of symptoms and signs related to the DAA and TOF. The pulse oximeter oxygen saturation on air was 96%. And follow-up echocardiograms have revealed satisfactory flow into the reconstructed pulmonary artery and without any gradients to descending aorta. The pressure gradient across pulmonary artery decreased to 16 mmHg after operation.

## Conclusions

DAA is a form of vascular rings, completely encircling trachea and esophagus by its connecting segments, which may cause stridor, respiratory distress or dysphagia in the first months of life [1]. During embryogenesis a failure of the normal regression of one or more segments of the six pairs of the aortic arches that arise from the truncus arteriosus leads to the formation of multiple anomalies of the aortic arch, to form DAA [2]. The vascular ring anomaly of the aortic arch comprises 1% of operable congenital cardiac disease [3]. In DAA patients, right or left arch may be larger or similar in size, or one arch can be atretic. But in clinic practices, right arch is dominant in more than 75% of these cases. In the present case, the patients had a DAA with a non-dominant left arch. DAA has been abundantly described in previous studies and its management is well established [4,5].

TOF is one of common cyanotic congenital heart disease that occurs in approximately 1 in 3600 live births and accounts for 3.5% of infants born with congenital heart disease. Surgical repair was first introduced in the 1950s, and nowadays most patients with TOF can undergo compete repair with a low operative mortality and a excellent long-term survival rate [6,7].

For patients with TOF, exertional dyspnoea is a common symptom. And DAA with mild stridor or dysphagia or without might be undetected preoperatively. So it is important for echocardiographic investigation to recognize before operation. A suprasternal fossa short-axis and long-axis view of aorta is necessary for echocardiographic investigation to find DAA anomalies. Once a suspected DAA is detected by echocardiography, a CT or cardiac catheterization check is vital for confirming anomalies and surgery treatment.

## Consent

Both patients have given their consents for the case report to be published, and written informed consents were obtained from the patients for publication of this case report and any accompanying images.

## Abbreviations

CT: Computed tomography; DAA: Double aortic arch; TOF: Tetralogy of Fallot; VSD: Ventricular septal defect

## Competing interests

The manuscript has not been submitted to nor is it under consideration for publication by another journal. None of the authors has any conflict of interest in the matter.

## Authors’ contributions

AL, GL, YL,and YbZ have participated in executing the work, collecting the data, writing of the manuscript. ZqL and YlL conceived of the study, and participated in its design and coordination and helped to draft the manuscript. All authors read and approved the final manuscript.
